# High-power multi-megahertz source of waveform-stabilized few-cycle light

**DOI:** 10.1038/ncomms7988

**Published:** 2015-05-05

**Authors:** O. Pronin, M. Seidel, F. Lücking, J. Brons, E. Fedulova, M. Trubetskov, V. Pervak, A. Apolonski, Th. Udem, F. Krausz

**Affiliations:** 1 Ludwig-Maximilians-Universität München, Fakultät für Physik, Am Coulombwall, 85748 Garching, Germany; 2 Max-Planck-Institut für Quantenoptik, Hans-Kopfermann-Str. 1, 85748 Garching, Germany

## Abstract

Waveform-stabilized laser pulses have revolutionized the exploration of the electronic structure and dynamics of matter by serving as the technological basis for frequency-comb and attosecond spectroscopy. Their primary sources, mode-locked titanium-doped sapphire lasers and erbium/ytterbium-doped fibre lasers, deliver pulses with several nanojoules energy, which is insufficient for many important applications. Here we present the waveform-stabilized light source that is scalable to microjoule energy levels at the full (megahertz) repetition rate of the laser oscillator. A diode-pumped Kerr-lens-mode-locked Yb:YAG thin-disk laser combined with extracavity pulse compression yields waveform-stabilized few-cycle pulses (7.7 fs, 2.2 cycles) with a pulse energy of 0.15 μJ and an average power of 6 W. The demonstrated concept is scalable to pulse energies of several microjoules and near-gigawatt peak powers. The generation of attosecond pulses at the full repetition rate of the oscillator comes into reach. The presented system could serve as a primary source for frequency combs in the mid infrared and vacuum UV with unprecedented high power levels.

## Abstract (web summary)

Frequency combs have revolutionized the study of electronic structures and dynamics of matter but currently used lasers systems are limited in terms of achievable pulse energies. Here, Pronin *et al.* demonstrate few cycle pulse emission from a thin-disk laser with 150 nJ pulse energy and 7.7 fs pulse duration.

## Graphical abstract

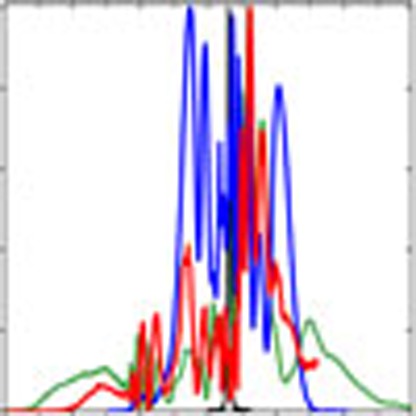

Femtosecond titanium-doped sapphire (Ti:Sa) laser oscillators and erbium/ytterbium fibre oscillators constitute a key workhorse of cutting-edge ultrafast science and precision spectroscopy. Their importance lies in the combination of a spectral output spanning up to an optical octave with the capability of achieving full control over the generated waveforms or, equivalently, the emitted frequency comb. These systems generally rely on additional amplifiers^1^ or passive enhancement cavities^2^ to boost the intensity of their pulses to levels allowing conversion to other wavelengths. In the frequency domain this approach allows the extension of stabilized frequency combs to short wavelengths, to reach the 1S-3S transition in atomic hydrogen at 205 nm (ref. 3) and enable the first direct frequency comb spectroscopy in this XUV region^4^, possibly enabling precision spectroscopy of hydrogen like He^+^ one day^5^.

In the time domain, the controlled mode-locked frequency comb grants access to the relative phase of the carrier wave with respect to the peak of the pulse envelope (carrier-envelope phase, CEP). This permits control over the electric field waveform. CEP-stabilized ultra-broadband Ti:Sa oscillators have enabled the generation of attosecond pulses^6^. Moreover, they have allowed control of electronic and subsequent nuclear motion in molecules^7^ and the generation of optical-field-induced currents in dielectrics^8^, with possibly far reaching implications to future high-speed metrology and signal processing^9^. Meanwhile, fibre oscillators with well-controlled CEP have become available and are rapidly proliferating in spectroscopy^10^.

However, due to the low output power and energy achievable directly from such laser oscillators (usually in the 100 mW average power, 1 nJ pulse energy range), most of these experiments have to rely on additional amplifiers or passive pulse-build-up (briefly: enhancement) cavities. As a consequence, the favourable properties of oscillators are compromised by adding system complexity, size, noise and—not least—cost. Furthermore, enhancing ultrashort pulses in passive-build-up cavities leads to a dramatic decrease in enhancement factors. Moreover, even amplified multi-kHz Ti:Sa systems provide insufficient data acquisition rates for several applications, such as cold target recoil ion momentum spectroscopy (COLTRIMS)^11^ and time resolved photoemission electron microscopy (PEEM).

All-solid-state, diode-pumped ytterbium-doped laser oscillators offer orders of magnitude higher average and peak powers. Among the several pump configurations, the thin-disk (TD) geometry appears to be most promising in terms of scalability to ultrahigh powers^12^,^13^. The development of TD oscillators has retraced the evolution of Ti:Sa oscillators. Energy scaling^14^,^15^,^16^, average power scaling^16^,^17^ and operation in the regime of normal dispersion^18^,^19^ have been realized. Although the first demonstration of a femtosecond TD oscillator dates back to the turn of the millennium^20^, applications in research or industry have not yet benefited from this technology having demonstrated the highest peak and average powers from mode-locked oscillators. This is partially due to the challenge of reliably maintaining saturable absorption mode-locking at kilowatt intra-cavity power levels^21^.

The recent demonstration of Kerr-lens mode-locked TD oscillators^16^,^22^,^23^ offers an alternative route towards reliable high-power femtosecond oscillator technology. Two more essential developments are left to be done to render TD lasers an order of magnitude more powerful alternative to Ti:Sa femtosecond oscillators CEP stabilization and few-cycle pulse operation.

The source reported here combines waveform control with high power and energy while retaining few-cycle operation. Excellent beam quality of the source allows focusing to a spot diameter of <3 μm, which implies peak powers on the order of 10^15^ W cm^−2^.

## Results

### Spectral broadening and pulse compression

**Figure 1:**
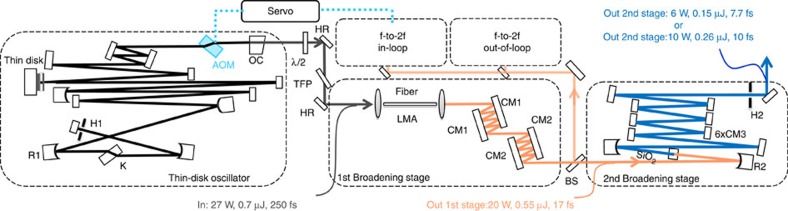
Experimental set-up of the 38 MHz laser system. Mode-locking of the oscillator was achieved using the Kerr lens action of a fused silica plate (K) and a hard aperture (H1). Sixty percent of its output power was picked by a half wave plate (*λ*/2) and a thin-film polarizer (TFP) and coupled into an LMA fibre for spectral broadening. Double-angle dispersive mirrors (CM1, CM2) were used to compress the pulses. One watt of this was used for CEO frequency detection in two identical interferometers. The error signal generated by a PLL scheme was fed back to an AOM operated as a loss modulator to control the pulses' CEP slip. In the second stage, the pulses were focused into a crystalline quartz plate (SiO_2_). Temporal compression to 9.9 fs was achieved by a complementary dispersive mirror set (CM3) and to 7.7 fs by two tailored double angle dispersive mirrors, followed by spatial filtering in an aperture (H2). The footprint of the whole system is 2.5 × 0.6 m^2^.

The relatively narrow bandwidth of TD laser gain media prohibits the generation of few-cycle pulses directly from a high-power oscillator. To overcome this intrinsic limitation, external spectral broadening and subsequent pulse compression can be utilized^24^,^25^. Our laser system consists of a KLM Yb:YAG TD oscillator^22^ and two broadening and compression stages (Fig. 1).

**Figure 2:**
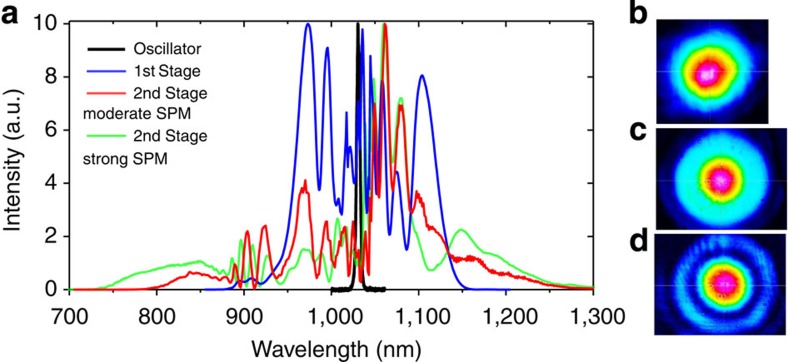
Spectra and beam profiles obtained in the two spectral broadening stages. (**a**) The oscillator provided Fourier-limited pulses with a bandwidth of 4.5 nm (black line). SPM in an LMA fibre led to a spectrum corresponding to a bandwidth limit of 14 fs (blue line). Further compression in bulk crystal quartz was strongly dependent on the crystal position with respect to the focal point: The red and green curves show spectra obtained with moderate and strong spectral broadening in the medium, supporting pulses down to 8 and 6 fs, respectively. (**b**) Output beam profile after first broadening stage, corresponding to the blue curve in **a**. (**c**) Output beam profile after bulk crystal, corresponding to the red curve in **a** and measured with 700-nm longpass filter. (**d**) Output beam profile after bulk crystal, corresponding to the red curve in **a** and measured with band pass filter at 1,064 nm. The beam profiles were measured with CCD camera sensitive in the range 300–1,100 nm.

The TD oscillator delivers 1.1-μJ, 250-fs pulses with an average power of 40 W (see Methods). The laser output is coupled into a large-mode-area photonic crystal fibre (PCF) of 26 μm mode field diameter and 8 cm length for spectral broadening via self-phase modulation. At an incident power level of 27 W, the PCF transmits an average power of 24 W. The resultant 85% coupling efficiency provides a clear indication of laser beam quality. To avoid damage of the input fibre facet, the peak power of the incident pulses is kept about 2.5 MW. The broadened output spectrum corresponds to a Fourier-limited pulse duration of 14 fs (Fig. 2a).

**Figure 3:**
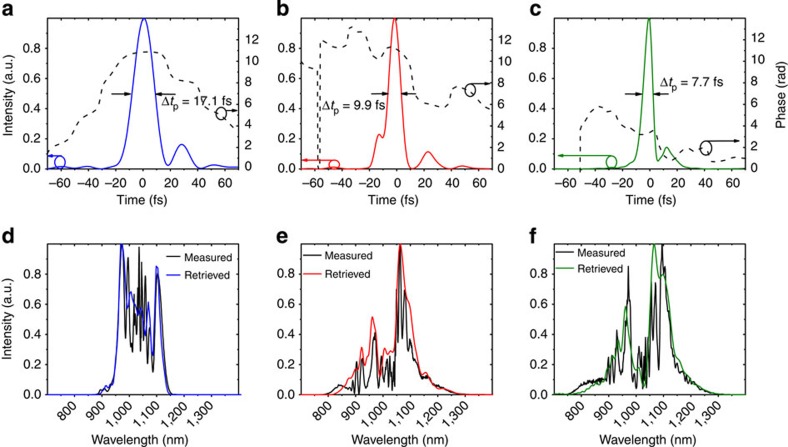
Pulse duration measurements. (**a**) Temporal intensity and phase of the pulses measured at the output of the first compression stage as retrieved by the FROG technique. About 85% of power is confined in the main 17.1 fs pulse. (**b**,**c**) Temporal intensities and phases of the pulses measured at the output of the second compression stage as retrieved by the FROG technique. About 75% of power is confined in the main 9.9 fs pulse. Approximately 80% of power is preserved in the main 7.7 fs pulse. (**d**–**f**) Corresponding retrieved spectral intensities in comparison with the spectra measured at the SHG crystal position with an OSA.

Temporal compression of the pulses was subsequently accomplished by two sets of dispersive mirrors. The pulse duration of 17 fs was measured by a frequency-resolved optical gating (FROG). The retrieved temporal intensity and phase from the FROG measurement are shown in Fig. 3a. Overall losses in the first compressor stage amounted to ∼25%, including coupling, polarization extinction in the fibre and transmission losses in the dispersive-mirror compressor. Within a single step, the pulses were compressed from a duration of 250 to 17 fs, which is shorter than the shortest ones reported from Yb-based systems so far^24^,^25^.

The peak power of the compressed pulses (25 MW) exceeded the critical self-focusing power of fused silica (≈4 MW). Subsequent spectral broadening of these compressed pulses was therefore implemented in a thin (5 mm) bulk medium^26^,^27^. The beam was focused with a spherical silver mirror (f=50 mm) to on-axis peak intensities of ∼10^12^–10^13^ W cm^−2^. Placing a crystalline quartz plate few mm behind the focal point caused moderate self-phase modulation and self-focusing in the nonlinear medium. The resulting spectrum corresponded to a Fourier limit of about 8 fs (red trace in Fig. 2a), with 60% of the energy carried in the central part of the beam (see Methods). Compression was performed by six bounces on a set of complementary dispersive mirrors with a total group delay dispersion (GDD) of −240 fs^2^ and third-order dispersion (TOD) of −480 fs^3^. Even broader spectra were obtained at the expense of efficiency (green trace in Fig. 2a) by moving crystal closer to the focal point. Compression down to 7.7 fs corresponding to 2.2 optical cycles was achieved with tailored dispersive mirrors. The pulses retrieved from SH-FROG measurements in the case of moderate and strong spectral broadening are shown in Fig. 3b,c, respectively.

### CEP characterization and stabilization

To investigate the CEP properties of the few-cycle source, the output of the first compression stage was sent to an f-to-2f interferometer (see Fig. 1). An octave-spanning spectrum was generated using a highly nonlinear PCF (see methods). First, we studied the carrier-envelope offset (CEO) frequency noise of the mode-locked laser without CEP stabilization. Owing to the strong amplitude-to-phase coupling induced in the Kerr-medium (1 mm fused silica plate)^28^, the CEO frequency sensitivity with respect to variation of the intra-cavity power was found to be as high as ≈20 MHz W^−1^ at an intra-cavity average power level of 280 W. This strong coupling permits CEP control with an extremely small modulation of the intra-cavity power, that is, without notably compromising the amplitude noise of the laser. The oscillator intensity noise was 0.3% r.m.s. (measured in the frequency range of 1 Hz to 500 kHz), a value comparable to state-of-the-art femtosecond Ti:Sa oscillators.

**Figure 4:**
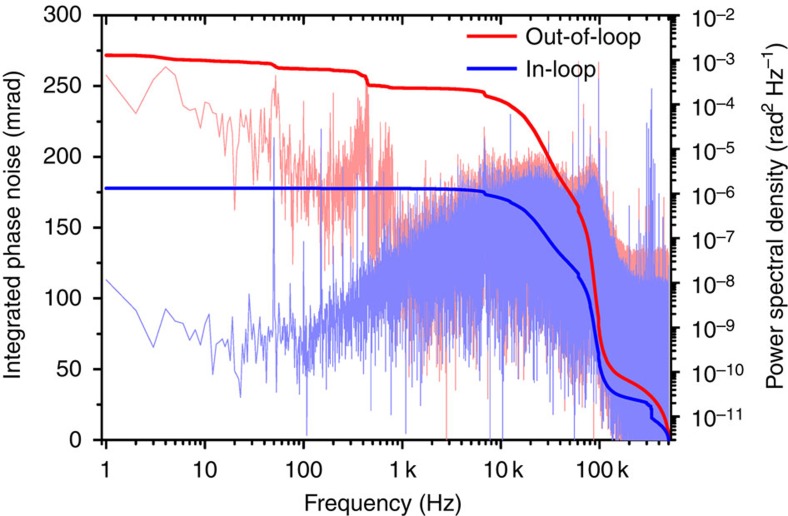
Residual CEO phase noise characterization. Power spectral density (light lines) and integrated noise (bold lines) of the residual phase error measured in the f-to-2f interferometers after the first compression stage. The in-loop measurement done with a digital phase detector with a 4-bit capture range is shown in blue, the out-of-loop measurement performed with an analogue phase detector in red. Differences between the two measurements stem from interferometer drifts and coupling into the highly nonlinear PCF (<1 kHz) as well as from the digital phase detector used within the PLL (>10 kHz). The integrated phase noise in-loop and out-of-loop amounts to 180 and 270 mrad, respectively.

Common methods of CEP stabilization rely on the intensity-dependent nonlinear phase shift experienced by the intra-cavity pulse^29^,^30^,^31^. By acting on intra-cavity energy, for example via the laser gain, the amount of nonlinear phase shift in each roundtrip can be controlled. However, any attempt at modulating the laser gain is effectively low-pass filtered with a time constant equal to the lifetime of the upper state of the lasing transition. Any noise above this frequency cannot be efficiently suppressed by gain modulation. In contrast to Ti:Sa oscillators with a gain relaxation time of about 3 μs, the millisecond response time of ytterbium-doped gain media restricts the bandwidth of the CEP control loop based on gain modulation to a few kHz, leaving sizeable noise uncompensated at higher frequencies. Using a home-built frequency discriminator, we determined the roll-off of the CEP slip frequency response to a modulation of the pump diodes. The measured 3 dB point was about 1.5 kHz, in accordance with a gain relaxation time of ∼1 ms, revealing a severe limitation in CEP control via laser gain. Therefore, we chose to resort to intra-cavity loss modulation, which has been demonstrated with absorptive graphene electro optic modulators in low power fibre oscillators^32^. This method offers the advantage of providing high loop bandwidth and independence of laser gain dynamics. Owing to the intracavity average power of 280 W, loss modulation had to be realized without any absorption in the stabilizing device. Therefore, to the best of our knowledge, CEP stabilization by means of an intra-cavity acousto-optic modulator (AOM) was realized for the first time. The AOM used has a thickness of ∼3 mm and was placed at Brewster angle inside the resonator (Fig. 1). It can introduce a loss modulation of up to 2%. The CEO frequency was locked to a radio frequency (RF) reference of 10.7 MHz using a phase-locked loop (PLL)^33^. The results are shown in Fig. 4. The in-loop measurement indicates a residual phase noise as low as 180 mrad r.m.s. (in the frequency band of 1 Hz–500 kHz), whereas the out-of-loop measurement yields an r.m.s. noise figure of ∼270 mrad. The difference of about 90 mrad is accumulated in two frequency ranges clearly discernible in Fig. 4. In the frequency band of 40–400 Hz, noise^34^ is supposed to be dominated by fluctuations in coupling losses into the nonlinear fibres. The increased out-of-loop noise level in the region between 10 and 100 kHz can be attributed to digitization noise in the phase detector employed in the PLL.

## Discussion

We conclude with a few comments on the power scalability of the new waveform-stabilized few-cycle laser technology demonstrated. CEP stabilization with an intra-cavity AOM can be used in a wide range of intra-cavity peak powers. Provided that the laser noise spectrum remains comparable, we expect that an increase of the intra-cavity peak power from currently 40 MW to more than 400 MW is possible without compromising stabilization performance. Spectral broadening in solid-core PCFs is average power scalable^35^, which is beneficial for frequency domain applications, but only moderately pulse energy-scalable^36^, which is desirable for time domain applications involving extreme nonlinear optics. Hence, it can hardly be employed in broadening experiments with the latest mode-locked TD laser generation^15^,^16^. Spectral broadening in multiple bulk crystals or broadband hollow-core Kagome fibres developed recently^37^,^38^,^39^ may constitute a viable alternative.

In summary, we have demonstrated CEP stabilization of a KLM TD oscillator with a residual phase noise of 270 mrad (π/12) r.m.s. measured out-of-loop within a bandwidth of 1 Hz–500 kHz. The reported source delivers 7.7-fs pulses, which contain 2.2 optical cycles within their FWHM and constitute the shortest pulses produced from TD oscillators so far. The source reported here delivers an order of magnitude higher average power and pulse energy than any other CEP-stabilized oscillator demonstrated so far, opening new prospects for few-cycle nonlinear optics, as well as attosecond physics and precision metrology.

## Methods

### Kerr-lens mode-locked thin-disk oscillator

The oscillator was built around an Yb:YAG thin-disk laser head. The disk was water-cooled from the back side and pumped by 250 W of 940 nm light from fibre-coupled laser diodes. Mode-locking was achieved by Kerr-lens action combined with an aperture situated in front of the end mirror (M in Fig. 1). Pulse formation was started by moving a mirror placed on a translation stage (R1 in Fig. 1). Net negative dispersion was provided by several highly dispersive mirrors. Operated with a 14% output coupler, it delivered 1.1-μJ, 250-fs pulses with an average power of 40 W at a repetition rate of 38 MHz. Spectrum and temporal shape indicate sech^2^-shaped pulses characteristic of soliton mode-locking at a central wavelength of 1,030 nm. The laser is described in detail in ref. 22. As a means to control the intra-cavity energy and CEP, we placed an AOM in the laser cavity close to the output coupler, where the beam diameter was about 2 mm. At 6 W of RF input power, it introduced about 2% loss to the cavity mode while causing negligible loss when switched off due to operation at Brewster's angle. The comparatively narrowband intra-cavity spectrum of 4.5 nm FWHM was not sensitive to the dispersion introduced by the fused silica device. Moreover, the nonlinear phase shift was negligible in comparison with that induced by the Kerr medium. Thus, the oscillator performed identically with or without the AOM inside the cavity.

### Temporal compression

We chose a two-stage compression scheme to achieve few-cycle pulses from initially long output provided by the TD oscillator. In the first stage, an 8-cm long LMA PCF (LMA35, NKT Photonics) with a mode field diameter of 26 μm was employed. Owing to its large core size, the LMA 35 fibre exhibits the dispersion of fused silica (Supplementary Fig. 4). The waveguide dispersion is negligible. Longer fibres exhibited complete saturation of spectral broadening. Temporal compression was achieved by two sets of dispersive mirrors pairs (Supplementary Fig. 1) with combined GDD of ≈−2,100 fs^2^ and a TOD of ≈−1,100 fs^3^. The mirror pairs were designed for double-angle configuration^40^ to minimize GDD oscillations. Among the materials fused silica, sapphire, YAG and CaF_2_, crystalline quartz yielded the best results in the second compression stage with respect to thermal stability, beam quality and spectral broadening. By choosing the relatively short focal length of 50 mm, Gaussian beam divergence and self-focusing were balanced to keep the critical self-focusing length longer than the crystal thickness while maximizing self-phase-modulation. By changing the distance between focal point and quartz crystal, SPM and self-focusing were adjusted. A spectrum corresponding to a bandwidth-limited duration of 6 fs was obtained (green trace in Fig. 2a). The mirrors available for compression after the second stage had originally been designed to compensate the dispersion of fused silica and could not fully cancel the positive chirp after broadening in crystalline quartz. Especially in the case of strong broadening (green trace in Fig. 2a), this leads to notable satellite features in the compressed pulse (Fig. 3a–c).

**Figure 5:**
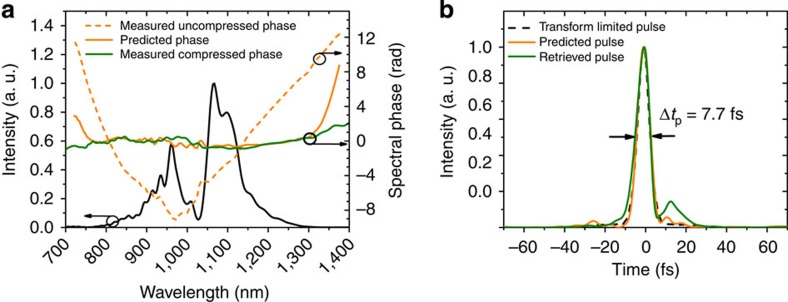
Pulse duration measurements. (**a**) Spectrum (black solid line) and spectral phase (green solid line) retrieved with FROG yielding the pulse shown in Fig. 3c. The uncompressed phase (orange dashed line) was also retrieved with FROG. It served as the target for the dispersive mirror design. The most prominent feature is the kink around 1,030 nm, which cannot be compensated by standard dispersive mirrors. The solid orange line is the sum of the uncompressed phase and the theoretical phase deduced from the dispersive mirror design. (**b**) The corresponding pulses for the spectrum shown in **a** and zero phase (dashed black line), the predicted phase (solid orange line) and the measured phase (solid green line). The excellent agreement between Fourier limited, predicted and measured pulse shows the capabilities of broadband dispersive mirror design and manufacturing as well as the phase sensitivity of the FROG measurements.

The pulse durations after the first and second stages were measured by a home-built dispersion-free second harmonic FROG apparatus with a 10-μm thick BBO crystal. In addition to every FROG measurement the fundamental spectrum was taken at the crystal position with an optical spectrum analyser (OSA, Ando) by focusing into a multi-mode fibre. The measured and the retrieved FROG spectra are shown in Fig. 3d–f, indicating reliable retrievals. The FROG error of the retrieved pulse after the fibre stage is 0.38% (512^2^ grid size). Despite the fairly high compression factor of about 15, the formation of a powerful pedestal can be excluded. This is shown in the Supplementary Fig. 3 by means of autocorrelation measurements and 1D simulations. The FROG error of the 9.9 fs pulse was 0.77% (512^2^ grid size) and 0.48% (512^2^ grid size) in the case of the shortest pulse. All FROG traces are shown in the Supplementary Fig. 2. The autocorrelator was not sensitive to small chirp variations in this case. Therefore, the device could not be used to cross-check the pulse duration. However, the phase sensitivity of the FROG device as well as the enormous capabilities of the latest dispersive-mirror design and fabrication technology are demonstrated by means of Fig. 5. To achieve clean compression of two-cycle pulses the uncompressed phase of the spectrally broadened pulses after the crystalline quartz plate was measured and fed into a multilayer design software ( http://www.optilayer.com/). Consequently, the optimized design was manufactured at our own facilities. Finally, the tailored mirrors were used for excellent compression of the ultrabroadband pulses.

### Spatial properties of the short pulses

In accordance with experiments performed earlier^26^,^27^, it was found that strong spectral broadening is accompanied with beam distortions due to spatial nonlinearities observed as a multiple rings around the central non-distorted part of the beam (Fig. 2d). In case of the 9.9 fs pulse the encircled power in the inner ring was about 60% of the incident power. In case of 7.7 fs pulse only about 40% of the energy was confined to the central part of the output beam. The central part of the beam was filtered out by the aperture and characterized with a scanning slit beam profiler (BeamScan, pyroelectric head). The M^2^ factor was measured along the *x*- and *y*-axis according to the ISO 11146 standard. The M^2^ factor of 1.3 after the fibre stage and 1.4 after the bulk stage were determined.

### CEP measurement and stabilization

**Figure 6:**
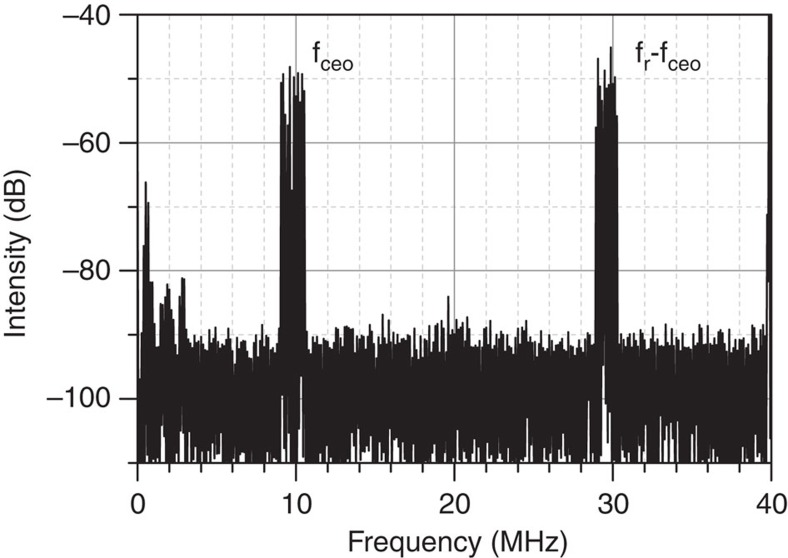
Free-running CEO frequency as measured with an RF spectrum analyser. The beat signal exhibits a signal-to-noise ratio of about 40 dB within a 10 kHz resolution bandwidth. f_ceo_, CEO frequency; f_r_, oscillator repetition rate.

For detection of the CEO frequency of the laser, we used two identical home-built f-to-2f interferometers in quasi-common-path geometry. To obtain octave spectral coverage, about 400 mW of average power was coupled into a 10-cm piece of highly nonlinear PCF (SC-3.7-975, NKT Photonics) in each interferometer. The typical signal-to-noise ratio was 40 dB (Fig. 6). The free-running CEO frequency exhibited large excursions spanning up to 4 MHz. We partially attributed these to the water cooling of the TD. Consequently, the water flow was decreased from 3 l min^−1^ down to 0.5 l min^−1^, suppressing higher frequency noise. Moreover, the adjustment of the distance of Kerr-medium (K in Fig. 1) from the focus was found to have a strong impact on the CEO behaviour and could be optimized to minimize intrinsic CEO fluctuations. To stabilize the CEO frequency in a PLL, the signal of one of the interferometers was bandpass filtered at 10.7 MHz, amplified and sent to one of the inputs of a ±32π digital phase detector (Menlo Systems). The reference signal was obtained from a stable RF generator and fed to the second input of the phase detector. The phase error was processed by a proportional-integral-derivative controller (D2-125, Vescent Photonics). The resulting servo signal was used as the amplitude modulation input of the AOM driver. We obtained optimal results with two integrators at 10 and 20 kHz corner frequency and a differentiator cut-on at 50 kHz.

## Author contributions

O.P., M.S. conceived the experiment. F.L. conceived preliminary spectral broadening experiments and beat signal detection, J.B. contributed in the oscillator development and maintenance. E.F., V.P., M.T. designed, manufactured and measured dispersive optics. A.A. and T.U. supervised CEP stabilization. F.K. initiated and supervised the study. All authors participated in the discussion of the manuscript.

## Additional information

**How to cite this article:** Pronin, O. *et al.* High-power multi-megahertz source of waveform-stabilized few-cycle light. *Nat. Commun.* 6:6988 doi: 10.1038/ncomms7988 (2015).

## Supplementary Material

Supplementary Information Supplementary Figures 1-4, Supplementary Methods and Supplementary References.
