# Supplementary material for: High-power multi-megahertz source of waveform-stabilized few-cycle light
Source: Nat Commun. 2015 May 5;6:6988. doi: 10.1038/ncomms7988 (PMC4432647; doi:10.1038/ncomms7988)
Supplement: Supplementary Information — Supplementary Figures 1-4, Supplementary Methods and Supplementary References. [file ncomms7988-s1.pdf]

Supplementary figures

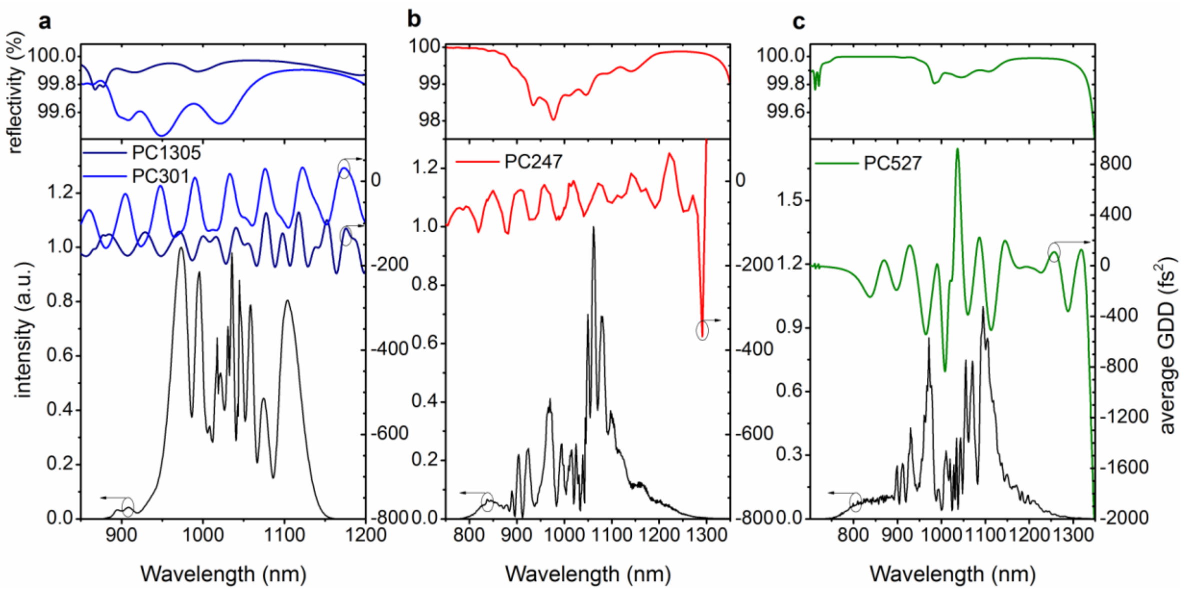

Supplementary Figure 1 | Spectra and dispersion-compensation curves. a, Spectrum after the first broadening stage in LMA 35 fibre. GDD curves of two mirror sets PC1305 and PC301 are shown in dark blue and blue, resp. Mirror set PC1305 has an average GDD per bounce of ≈150 fs^2 and mirror set PC301 has GDD≈60 fs^2 with TOD≈120 fs^3. **b,** Spectrum after the second broadening stage with moderate SPM. Mirror set PC247 has GDD≈40 fs^2 and TOD≈120 fs^3: **c,** Spectrum after the second broadening stage with strong spectral broadening and the tailored mirror set PC527 (in green) for dispersion compensation.

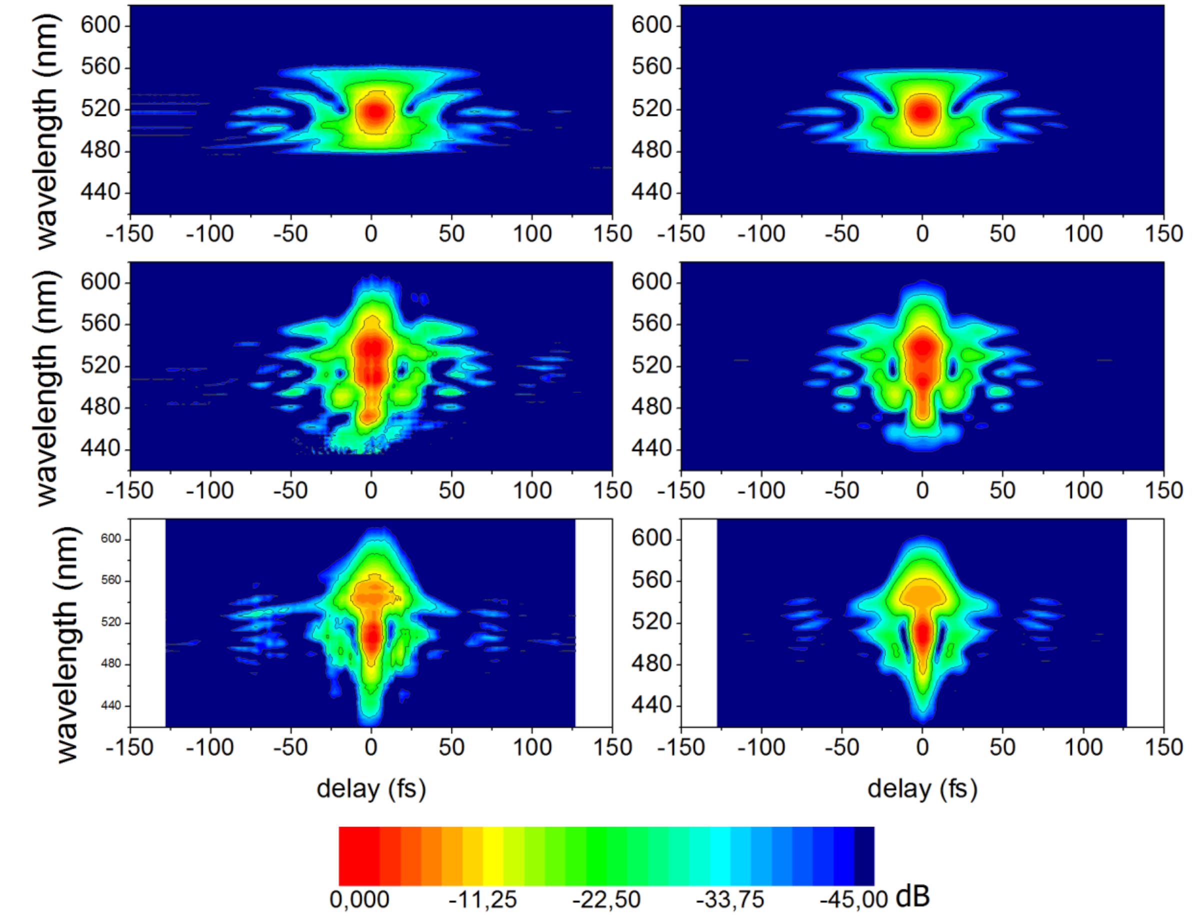

Supplementary Figure 2 | Measured and retrieved FROG traces a. measured FROG trace 17.1 fs pulse. **b**. retrieved FROG trace 17.1 fs pulse. **c**. measured FROG trace 9.9 fs pulse. **d**. retrieved FROG trace 9.9 fs pulse. **e**. measured FROG trace 7.7 fs pulse. **f**. retrieved FROG trace 7.7 fs pulse.

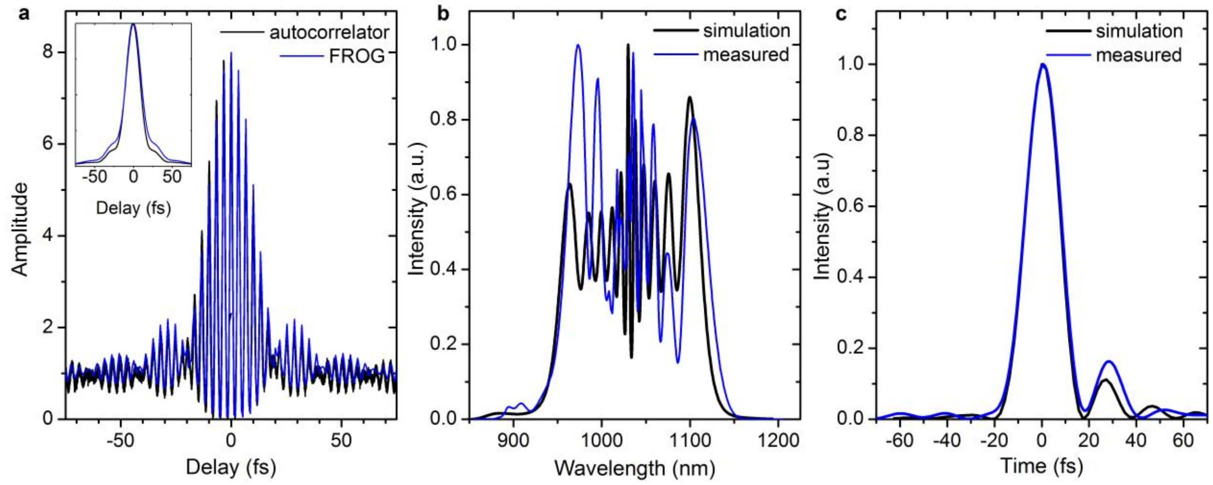

Supplementary Figure 3 | Cross-checks of the first spectral broadening and compression stage. a. comparison of measured and retrieved autocorrelations after the first compression stage. The inset shows the deduced intensity autocorrelations. The measured fringed resolved autocorrelation is slightly asymmetric due to the asymmetric beam splitter in the autocorrelator [SI1]. **b**. Comparison between simulated and measured spectra after 8 cm propagation in LMA 35. **c**. Comparison between measured pulse and the simulated pulse after compression with PC1305 mirrors.

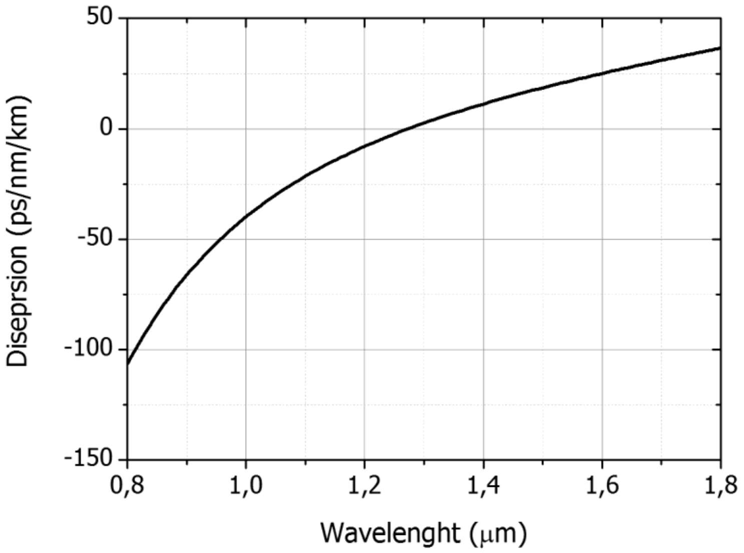

Supplementary Figure 4 | Dispersion of LMA 35 fibre. Due to its large core size, the LMA 35 fibre exhibits the dispersion of fused silica. The waveguide dispersion is negligible.

Supplementary methods.

Pulse compression. The chirped pulses after the broadening stages were compressed with different sets of dispersive mirrors[2]. The mirrors used after the first fibre stage (12 reflections on PC1305 and 8 reflections on PC301) required a double-angle configuration to effectively minimize GDD oscillations[3]. The mirrors PC247 (6 reflections) used for pulse compression after the bulk stage down to 9.9 fs had a complementary design[4,5]. Additional fine-tuning compression with PC527 mirrors (double-angle configuration, 2 reflections) allowed to obtain 7.7 fs pulses. The theoretical reflection and GDD curves shown in Fig. 1 always present average properties. All mirrors were measured prior to the experiment and agreed well with the design.

FROG measurements. The FROG setup was based on the second harmonic FROG setup presented in[6]. However, a wavefront division interferometer was utilized which only contained silver mirrors, i.e. no transmissive elements. The separated beams were noncollinearly focused with an off-axis parabolic mirror (EFL = 2”) into a 10 μm thick BBO-crystal to generate the delay dependent second harmonic. The FROG signal was separated from the other beams by an aperture placed 25 cm behind the crystal. The signal was coupled into a multimode fibre with a spherical focusing mirror and measured with an Ocean Optics HR4000 optical spectrometer (0.5 nm resolution). The delay was scanned by varying one interferometer arm length with a step motor (Zaber TN, 0.08 μm minimal step size).
In addition to every FROG measurement the fundamental spectrum was taken at the crystal position with an optical spectrum analyzer (OSA, Ando AQ-6315A) by focusing into a multimode fibre.

The measured and the retrieved FROG trace of the first stage are shown in fig. 2 a. and b., resp. The FROG error of the retrieved pulse is 0.38 % (512^2 grid size). Autocorrelation

measurements (Femotchrome FR-103PD) agreed well with the retrieved autocorrelation as shown in Fig. 3a. Pedestals for higher delays were not detected. This has also been proved by means of simulations[7] including SPM, self-steepening and Raman contributions as fibre nonlinearities. The simulations were performed with 550 nJ input energy which is slightly lower than in the experiment (595 nJ) and can be attributed to uncertainties in the exact mode field diameter of the fibre as well as the n_2 value at 1030 nm.

The second stage measurements are shown in Fig. 2c. – f. In the case of the 9.9 fs pulse (Fig. 2c. measured, Fig. 2d. retrieved FROG trace), the residual FROG error was 0.77 % (512^2 grid size). The FROG error of the 7.7 fs pulse was 0.48 % (512^2 grid size, Fig. 2e. measured, Fig. 2f. retrieved FROG trace).

The autocorrelator was not sensitive to small chirp variations in case of the very broadband pulses. Therefore, the device could not be used to cross-check the pulse duration. Instead, the phase sensitivity of the FROG was checked by adding a well defined amount of dispersion (i.e. by adding thin fused silica plates) and retrieving this phase variations by additional FROG measurements.

Supplementary references

1. Spielmann, C., Xu, L. & Krausz, F. Measurement of interferometric autocorrelations: comment. *Appl. Opt.* **36,** 2523-2525 (1997).
2. Szipöcs, R., Spielmann, C., Krausz, F. & Ferencz, K. Chirped multilayer coatings for broadband dispersion control in femtosecond lasers. *Opt. Lett.* **19,** 201-203 (1994).
3. Pervak, V., Ahmad, I., Trubetskov, M.K., Tikhonravov, A.V. & Krausz, F. Double-angle multilayer mirrors with smooth dispersion characteristics. *Opt. Express* **17,** 7943-7951 (2009).
4. Kärtner, F.X. *et al.* Ultrabroadband double-chirped mirror pairs for generation of octave spectra. *J. Opt. Soc. Am. B* **18,** 882-885 (2001).
5. Pervak, V. *et al.* 1.5-octave chirped mirror for pulse compression down to sub-3 fs. *Appl. Phys. B* **87,** 5-12 (2007).
6. Trebino, R., *Frequency Resolved Optical Gating*. (Kluwer Academic Publishers, 2000).
7. http://www.fiberdesk.com.
